# Inflammatory Lymphedema Masquerading as Bilateral Cellulitis: A Military Dilemma

**DOI:** 10.7759/cureus.51743

**Published:** 2024-01-06

**Authors:** Caroline E Moore

**Affiliations:** 1 Internal Medicine, Naval Medical Center San Diego, San Diego, USA

**Keywords:** skin condition, antibiotic stewardship, prolonged standing, military medicine, bilateral cellulitis, military recruit, inflammatory lymphedema, leukocytoclastic vasculitis

## Abstract

Bilateral lower extremity inflammatory lymphedema (BLEIL) is a novel condition characterized by confluent erythema and painful edema prominent to the dorsum of the feet and ankles bilaterally following prolonged standing and marching, occurring most often in military recruits. Prolonged standing during the initial week of basic training is thought to cause venous congestion and subsequent inflammatory vasculitis. This condition may be misdiagnosed as bilateral cellulitis, prompting the initiation of unnecessary antibiotic therapy. Increased education and recognition of this new clinical entity would lead to the initiation of appropriate therapy and earlier symptom resolution and, thus, an earlier return to military training.

Herein, we describe a small case series of Marine Corps recruit members undergoing their first week of basic training (i.e., "processing week") who developed bilateral lower extremity edema, erythema, and pain localized to the dorsum of the feet and the medial and lateral aspects of the ankles consistent with the diagnosis of BLEIL but were initially diagnosed with bilateral lower extremity cellulitis and received intravenous antimicrobial therapy. With prompt initiation of venous decongestive therapy with leg elevation, both patients had rapid symptom resolution and returned to basic training without any future episodes of symptoms. These cases add to the paucity of data on this clinical entity, illustrate the symptoms and demographics of BLEIL, and describe the importance of recognition and initiation of appropriate therapy.

## Introduction

Bilateral lower extremity inflammatory lymphedema (BLEIL) is a novel condition, only recently recognized in 2015, characterized by the rapid development of edema, erythema, and excruciating pain involving the dorsum of the feet and ankles. Unfortunately, the incidence and the demographic distribution of this diagnosis have not been well described due to the paucity of data, but the vast majority of cases have occurred in male, physically fit recruits [1]. From the limited available data, these patients often receive antimicrobial therapy at first, as cellulitis is often an initial diagnostic consideration [1,2]. Importantly, however, patients with BLEIL have a bilateral distribution, a lack of disruption of the epidermis, and hyperacute onset of symptoms, which differentiate this diagnosis clinically from cellulitis. Additionally, bilateral cellulitis is extremely rare [3]. However, as these signs and symptoms may bear a resemblance to acute cellulitis, the initiation of unnecessary antimicrobial therapy is common, and this clinical entity is likely misdiagnosed and underrecognized [1,2]. This ultimately leads to diagnostic delay and a delay in initiating appropriate treatment. This diagnosis has been recently identified as developing in military recruits across all services, and symptoms are thought to develop after prolonged standing (i.e., standing at attention), which ultimately causes leukocytoclastic vasculitis [1,4]. Fortunately, this diagnosis is self-limiting with rapid resolution with decongestive therapy and rest. 

It is important to recognize the context of the development of this condition, as it may aid in a quicker diagnosis. BLEIL has been described to almost always occur during the first week of recruit basic training, referred to as "processing week", where recruits are standing for prolonged hours and marching from department to department for administrative check-ins. Specifically, there is no other physical fitness occurring outside of prolonged standing. However, there has been a reported case occurring in a marathoner, and thus, this diagnosis may affect more individuals than just military recruits [2]. The cases presented herein highlight the clinical presentation of this novel diagnosis as well as emphasize the importance of differentiating BLEIL from bilateral cellulitis, which is a very rare entity, to ensure avoidance of a diagnostic delay and engagement in the correct mode of therapy. This case also serves to add to the paucity of data available on this diagnosis as well as raise awareness of this diagnosis to both military and civilian providers who may provide care to military members over the course of their career or may encounter this diagnosis in civilian athletes in the future.

## Case presentation

Case 1

A previously healthy 23-year-old Caucasian male, on the fifth training day of Marine Corps recruit training, presented to the emergency department (ED) for acute onset of bilateral lower extremity edema and erythema with proximal progression. These symptoms started one day prior, and he also reported severe tenderness to palpation of the affected areas on his bilateral lower extremities. The marine recruit described extended periods of prolonged standing at attention since the onset of training five days prior, with frequent marches of several miles, and it had become quite difficult to put on his required footwear. His symptoms started as mild discomfort at the ankles, with rapid progression of severe pain and swelling. He denied any skin breakdown, cuts, or points of possible microbial entry on his lower extremities or toes. Additionally, he denied fevers, chills, night sweats, upper respiratory symptoms, cough, orthopnea, abdominal pain, nausea or vomiting, tick or insect bites, or changes in bowel movements. He denied a history of similar episodes. The patient had no past medical history and was not taking any medications. He denied any alcohol, illicit drug, or tobacco product use. There was no significant family history. On physical examination, his vital signs were within normal limits besides a non-sustained low-grade maximum temperature of 100.3 degrees Fahrenheit. He was a physically fit male (body mass index of 18) in no acute distress with a regular heart rate and regular rhythm, and his lungs were clear to auscultation. There was no jugular venous distention (JVD) and no palpable lymphadenopathy, but he did have one-millimeter (mm) pitting edema of the bilateral lower extremities to the level of the malleoli bilaterally. The pitting edema was most prominent at the dorsum of both feet with extension to the medial aspect of ankles. The area was associated with exquisite tenderness to palpation, and the extremities were mildly warmer than other uninvolved areas of the integument. Additionally, he had mild, confluent erythema at the dorsum of both feet and the lateral and medial malleoli. He had no petechiae or purpura involving his integument. The rest of his examination was unremarkable. His initial laboratory evaluations ordered by the ED provider are listed in Table 1. His urinalysis was bland.

**Table 1:** Initial emergency department laboratory parameters of case 1 cells/mm3 - cells per cubic millimeters; g/dL - grams per deciliter; cells/uL - cells per microliter; mmol/L - millimoles per liter; mg/dL - milligrams per deciliter; mcg/L - micrograms per liter

Parameter	Observed value	Normal range
White blood cells (WBC)	9500 cells	3200-10,800 cells/mm^3^
Hemoglobin	10.9 g/dL	13.1-18.6 g/dL
Platelets	147,000 cells/uL	150,000-350,000 cells/uL
Serum sodium	137 mmol/L	136-145 mmol/L
Serum potassium	3.8 mmol/L	3.5-5.1 mmol/L
Serum chloride	102 mmol/L	98-107 mmol/L
Serum bicarbonate (HCO3-)	23 mmol/L	21-32 mmol/L
Serum urea	11 mg/dL	7-18 mg/dL
Serum creatinine	1.0 mg/dL	0.7-1.3 mg/dL
Random blood glucose	100 mg/dL	70-100 mg/dL
Serum calcium	8.2 mg/dL	8.5-10.1 mg/dL
Serum phosphorus	2.7 mg/dL	2.5-4.9 mg/dL
Serum magnesium	2.4 mg/dL	1.6-2.6 mg/dL
Serum ferritin	161 mcg/L	24-336 mcg/L

The patient did receive one dose of intravenous (IV) ceftriaxone in the ED prior to consulting the admitting internal medicine (IM) service, as the ED team was concerned about bilateral lower extremity cellulitis. The marine recruit was admitted to the inpatient service as the recruit depot did not have the infrastructure in place to care for him. Given that the patient was well-appearing, hemodynamically stable, and his history and physical were inconsistent with cellulitis, the clinical decision to withhold further antibiotics was made. The clinical diagnosis of bilateral lower extremity inflammatory lymphedema (BLEIL) was made. The treatment focus was on leg elevation to the level of the heart and pain control. The patient was given venous thromboembolism (VTE) prophylaxis throughout his hospital stay. Within 48 hours, the recruit's symptoms had completely resolved without antimicrobial therapy, and his blood cultures had remained negative. Additionally, his hemoglobin increased to within normal limits, and his ferritin had downtrended on discharge, clinically resembling an anemia of inflammation. He was discharged back to the Marine Corps recruit depot to engage again in basic training. The recruit did not have a recurrence of symptoms. 

Case 2

An 18-year-old previously healthy male, on the seventh training day of Marine Corps recruit training, presented to the ED for acute onset of bilateral lower extremity edema and erythema. He described the proximal spread of his symptoms over the last day. The recruit stated that he noticed increased swelling, warmth, and erythema of both his lower extremities, beginning on the dorsum aspects of his feet, which had progressed to involve aspects of his ankles and bilateral shins. He also reported prolonged standing since entering training with infrequent changes of his socks and footwear. He denied any lacerations or breakdown of the skin on his legs or the existence of lesions between his toes. He denied fevers or night sweats but noted a feeling of chills. There was no history of tick bites, conjunctival injection, pharyngitis, cough, rhinorrhea or nasal congestion, shortness of breath or orthopnea, abdominal pain, or nausea or vomiting. He denied a history of similar episodes. The recruit had no previous medical history and was not taking any medications or over-the-counter supplements. He denied any history of allergies. He denied tobacco product use, alcohol, or recreational drug use. There was no significant family history. On physical examination, his vital signs were within normal limits, and his maximum temperature was 98.8 degrees Fahrenheit. He was a physically fit male (body mass index of 20) in no acute distress with a regular heart rate and regular rhythm, and his lungs were clear to auscultation. There was no JVD or palpable lymphadenopathy, but he did have 2 mm pitting edema of the bilateral lower extremities, most prominent at the dorsum of both feet with extension proximally to the ankles and shins bilaterally. He had a circumferential distribution of erythema associated with warmth to the same affected area. He had no petechiae or purpura involving his integument. Otherwise, the exam did not have any other remarkable findings. His initial laboratory evaluations ordered by the ED provider are listed in Table 2. His urinalysis was bland.

**Table 2:** Initial emergency department laboratory parameters of case 2 cells/mm3 - cells per cubic millimeters; g/dL - grams per deciliter; cells/uL - cells per microliter; mmol/L - millimoles per liter; mg/dL - milligrams per deciliter

Parameter	Observed value	Normal range
White blood cells (WBC)	11,400 cells	3200-10,800 cells/mm3
Hemoglobin	12.2 g/dL	13.1-18.6 g/dL
Platelets	186,000 cells/uL	150,000-350,000 cells/uL
Serum sodium	133 mmol/L	136-145 mmol/L
Serum potassium	3.5 mmol/L	3.5-5.1 mmol/L
Serum chloride	95 mmol/L	98-107 mmol/L
Serum bicarbonate (HCO3-)	24 mmol/L	21-32 mmol/L
Serum urea	11 mg/dL	7-18 mg/dL
Serum creatinine	1.1 mg/dL	0.7-1.3 mg/dL
Random blood glucose	94 mg/dL	70-100 mg/dL
Serum calcium	9.0 mg/dL	8.5-10.1 mg/dL
Serum phosphorus	2.4 mg/dL	2.5-4.9 mg/dL
Serum magnesium	2.0 mg/dL	1.6-2.6 mg/dL

No imaging was performed. Before consulting the admitting IM service, the ED had administered one dose of IV ceftriaxone for a possible diagnosis of bilateral lower extremity cellulitis. The patient was admitted as the infrastructure of the recruit depot did not have a place to care for him. As the patient was afebrile, relatively well-appearing, hemodynamically stable, and had a bilateral nature to his symptoms, the decision to withhold further antimicrobial therapy was made as the clinical suspicion for bilateral cellulitis was very low. There was a higher suspicion for an inflammatory etiology from gravity-dependent venous congestion, given the history and physical. Thus, the diagnosis of BLEIL was made, and the treatment plan focused on decongestive therapy and lymphatic drainage with elevation of the lower extremities to the level of the heart. Additionally, the marine recruit received VTE prophylaxis throughout his hospital stay. Within 48 hours, the patient's symptoms had completely resolved without additional antibiotic therapy, his leukocytosis resolved, and his blood cultures remained negative. He was discharged back to the Marine Corps recruit depot to engage again in basic training. He did not have a recurrence of his symptoms.

## Discussion

Bilateral lower extremity inflammatory lymphedema (BLEIL) is a novel condition first described in 2015 in Air Force trainees that has the clinical features of symmetric, confluent erythema, edema, and exquisite tenderness to palpation most prominently at the dorsum of the feet and the medial and lateral aspects of the ankles bilaterally [1]. This newly recognized condition is often misdiagnosed as acute cellulitis as there is a clinical overlap between the two diagnoses, and thus, patients often receive unnecessary antimicrobial therapy. However, bilateral cellulitis is an exceedingly rare clinical entity, and bilateral involvement should prompt a lower clinical suspicion of cellulitis in a patient [3]. BLEIL is thought to occur from prolonged venous congestion and perhaps due to a temporary venous pump failure from having a prolonged extension of the knees leading to interstitial edema and leakage of cytokines in the interstitial space to cause an inflammatory leukocytoclastic vasculitis (LCV) [1,2,4]. The literature describes histopathology as a deep dermal neutrophilic infiltrate surrounding the deep vascular plexus suggestive of an early LCV [4]. The patients described in this small case series are meant to illustrate and educate on the demographic and clinical symptoms of BLEIL cases comparatively to other diagnoses and to increase the recognition of this diagnosis among clinicians to avoid unnecessary antimicrobials and begin effective treatment earlier. 

BLEIL is a recently recognized diagnosis and, as such, may not be well known among civilian or military-trained physicians. Almost all of the cases that have been described in the literature have occurred in physically fit and/or thin military recruits during the first week of basic training, which is different from stasis dermatitis, which is chronic in nature and primarily affects those in middle age or above [5]. Specifically, it has been described to occur most often within the first 120 hours of arrival to basic training, which is consistent with our cases. From a review of the available literature, the majority of cases of BLEIL have occurred in males and in the age demographic of 18-23 years of age. An incidence was reported in one study as 0.4% among their population, but this may be an underestimation as the diagnosis is universally underrecognized given the lack of awareness of the diagnosis and the frequent misdiagnosis of bilateral cellulitis [1]. Importantly, it is crucial to recognize the population and context in which this clinical entity has been described to heighten suspicion among clinicians. The majority of cases have been recognized in new military trainees that arrive to recruit basic training and occur during the first week, which is described primarily as a "processing week", which includes recruits standing in line or at attention for hours as they march between the required departments upon check-in (i.e., uniform distribution, identification card distribution, medical clinic, military pay, etc.). Physically, the recruits are largely engaging in prolonged standing and marching the first week without any other forms of physical fitness. However, this diagnosis has also been reported in an ultramarathoner [2], and more studies are required to ascertain the full demographic affected. The key clinical symptoms of this diagnosis are confluent, mild erythema, pitting edema of the dorsum of the feet and the ankles bilaterally, as well as exquisite tenderness of the affected areas which is consistent with our two cases. Additionally, patients may have a mild leukocytosis, which improves rapidly following treatment. The results of the metabolic panels were within reference ranges. 

It's crucial to differentiate BLEIL from other diagnoses that may have clinical overlap, as this will lead to the quicker initiation of appropriate therapy. Importantly, bilateral involvement clinically differentiates this diagnosis from cellulitis, as bilateral cellulitis is exceedingly rare [3], which is a critical distinction to make to avoid unnecessary antimicrobial use and to practice antibiotic stewardship. Additionally, BLEIL needs to be differentiated from golfer's vasculitis (GV) or exercise-induced vasculitis (EIV). GV can also present with erythema and edema of the lower legs after prolonged standing or activity. However, GV has patchy erythema and mild, painless edema with the subsequent development of a purpuric rash, which is absent in BLEIL. Additionally, GV/EIV most often occurs in golfers after playing 18 holes, in older adults, and occurs almost exclusively in hot weather [2,4]. The lesions generally resolve spontaneously after about 10 days [6]. In contrast, the edema in BLEIL is exquisitely tender, lacks any purpuric lesions or petechiae, occurs in young, healthy adults, and resolves within 96 hours after leg elevation [1]. Furthermore, stasis dermatitis may be on the differential when evaluating a patient with BLEIL as stasis dermatitis can present as bilateral, pitting edema. However, stasis dermatitis is typically painless and due to chronic venous insufficiency with symptoms occurring over many years, which can cause hyperpigmentation of the skin of the lower extremities, which differentiates it from the acute process of BLEIL [7]. 

Recognizing BLIEL is critical to implementing appropriate treatment and avoiding unnecessary workup and antibiotic use. The treatment of BLEIL is primarily initiating venous decongestive therapy through leg elevation to the level of the heart and progression of ambulation as tolerated. Antimicrobial therapy has not been shown to improve symptom resolution [1]. After appropriate therapy with leg elevation, symptoms resolve rapidly, with most cases having complete resolution within 96 hours [1,2]. The patients described in this case report had symptom resolution within 48 hours. This diagnosis appears to be self-limiting from the available literature, which is consistent with our two cases [1,2,4]. Our patients did not have a recurrence of symptoms during the duration of their basic training when the electronic medical record (EMR) was reviewed retrospectively, and no other cases in the literature have reported a recurrence of symptoms [1,2]. 

The two cases herein illustrate the manifestations, demographics, and treatment for bilateral lower extremity inflammatory lymphedema to educate on this newly identified condition to add to the paucity of data and raise awareness for clinicians that practice in both the military and civilian sectors, as many of our military recruits have sought medical attention at civilian hospitals in the past. This will help to raise recognition of this condition to aid in clinical reasoning in the right demographic, but also in other populations where this diagnosis may be underrecognized (i.e., ultramarathoners and other occupations requiring long hours on their feet). It's postulated that this diagnosis may be more common than previously described (and likely misdiagnosed as cellulitis) in occupations requiring prolonged standing, although this is speculative. These cases may also provide clinicians with guidance on the approach to patients with BLEIL and reassurance on withholding antimicrobials to focus on venous decongestive therapy as the primary modality of treatment. Furthermore, earlier recognition of this diagnosis and implementation of leg elevation will avoid unnecessary antimicrobials without benefit and provide a more rapid return to training in military basic training. Further studies are needed to properly identify risk factors, possible preventative measures, incidence, prevalence, and the demographic of this clinical entity, as well as to define the risk of undiagnosed BLEIL in the military.

## Conclusions

In conclusion, this small case series significantly adds to the paucity of data on a novel condition occurring in a specific subset of the population, namely military recruits with normal body mass indexes, and highlights that although there is clinical overlap in BLEIL and acute cellulitis, the bilateral and symmetric nature, timing of symptom development, and lack of disruption of the epidermis are key differentiators of the diagnoses. These cases also emphasize the importance of making this differentiation to practice antibiotic stewardship, avoid unnecessary side effects of antimicrobial therapy and unnecessary medical workups, and begin effective treatment (i.e., elevation of the lower extremities), which rapidly improves symptoms. Furthermore, our cases highlight the clinical symptoms of BLEIL, which may also be present in other physically active groups in the civilian population outside of the military. Finally, with many of our military recruits seeking care in both the military and civilian sectors, it is crucial for both military and civilian providers to be aware of this novel condition so that basic trainees can return to the required training earlier. Future research is required to better understand, prevent, and manage this condition. 

## References

[1] Fajardo KA, Keller P, Kobayashi T, Hivnor CM, Webber BJ, Federinko SP, Tchandja J (2015). Bilateral lower extremity inflammatory lymphedema in Air Force basic trainees: clinical and epidemiologic study of a new disease entity. JAMA Dermatol.

[2] Robinson WH, Willardson HB, Nye NS (2023). Bilateral lower extremity inflammatory lymphedema after an ultramarathon. JAAD Case Rep.

[3] Raff AB, Kroshinsky D (2016). Cellulitis: a review. JAMA.

[4] McCann SE, Dalton SR, Kobayashi TT (2017). Histopathology of bilateral lower extremity inflammatory lymphedema in military basic trainees: a leukocytoclastic vasculitis of the deep vascular plexus. J Cutan Pathol.

[5] Falagas ME, Vergidis PI (2005). Narrative review: diseases that masquerade as infectious cellulitis. Ann Intern Med.

[6] Espitia O, Dréno B, Cassagnau E (2016). Exercise-induced vasculitis: a review with illustrated cases. Am J Clin Dermatol.

[7] Keller EC, Tomecki KJ, Alraies MC (2012). Distinguishing cellulitis from its mimics. Cleve Clin J Med.

